# Rare disease clinical trials in the European Union: navigating regulatory and clinical challenges

**DOI:** 10.1186/s13023-024-03146-5

**Published:** 2024-07-31

**Authors:** Sangita Mishra, MP Venkatesh

**Affiliations:** 1 Dept. of Pharmaceutics, Centre of Excellence in Regulatory Sciences, JSS College of Pharmacy, JSS Academy of Higher Education and Research, SS Nagara, Mysore, Karnataka 570015 India; 2 Faculty of Pharmaceutical Sciences, UCSI University, Kuala Lumpur, Malaysia

**Keywords:** Rare diseases, Orphan drugs, Clinical trials, Novel methodologies, Umbrella trials, Basket trials, Adaptive trials, Decentralized trials, Real world evidence

## Abstract

**Background:**

Clinical development for orphan drugs presents significant difficulties and challenges. There is no unique or standard design, conduct, and outcome assessment methodology and it is sometimes impractical to fit design models of rare disease trials in any practiced and well-known framework. In the European Union (EU) these challenges encompass a broad array of subjects, including trial design, study outcomes, patient recruitment, trial conduct ethics, trial cost, and chances of success. This literature-based review study aims to provide a thorough overview of the critical aspects of rare disease trials in the EU by analyzing the current landscape of rare disease trials, highlighting key challenges, delving into regulatory and research initiatives and innovation in trial designs, and proposing multi-faceted solutions to implement effective rare disease clinical trials in the region.

**Discussion:**

Traditional clinical trial designs, validation, and evaluation methodologies used for nonorphan drugs often prove unsuitable for orphan drugs, given the small patient populations, sometimes fewer than 1000 cases. There is an increasing need for accessible therapies and both regulators as well as industry are trying to develop affordable and effective drugs to address this need. Despite several steps that have been taken, the timely development of drugs remains a challenge. One of the reasons behind the long development timeline is the recruitment, retention, and conduct of rare disease trials. To optimize the development timelines of orphan drugs in the EU, it is important to ensure that the safety and efficacy of the product is not compromised. Industry and regulatory agencies must implement innovative trial designs, devise flexible policies, and incorporate real-world data for assessing clinical outcomes.

**Conclusion:**

Collaboration among academic institutions, pharmaceutical companies (both small and major), patient groups, and health authorities is crucial in overcoming obstacles related to clinical trials and providing assistance and creative ideas. The ultimate objective of granting rare disease patients timely and affordable access to medications with a positive balance between benefits and risks is to be met.

**Supplementary Information:**

The online version contains supplementary material available at 10.1186/s13023-024-03146-5.

## Background

Developing effective treatments for rare diseases is challenging due to their low prevalence in the EU, with only 5 patients per 10,000. Most rare diseases lack adequate treatment options. Conducting clinical trials with small populations is challenging resulting in limited evidence generation. To address this issue, regulatory guidance, such as the “Guideline on clinical trials in small populations” [1] by the European Medicines Agency (EMA) guidance in the EU, covers various aspects of clinical trials, including pharmacological factors, endpoint selection, control group selection, methodological considerations, statistical considerations, and levels of evidence. In 2012, the European Commission (EC) launched a call for proposals, “New methodologies for clinical trials for small population groups,” under the FP7 health innovation framework [2]. This initiative aimed to develop improved statistical methods for assessing the safety and effectiveness of treatments for small population groups, focusing on rare diseases and personalized medicine and increasing evidence generation from the clinical trials to support safety and efficacy outcomes. The objective was to reduce trial design costs and conduct effective clinical trials that produce reliable outcomes for rare disease studies involving small patient populations [3]. Randomized clinical trials have been tried on rare disease trials in the EU, but since the population of patients is small the question of appropriate sample inclusion and impact of outcomes of these trials in the long run cannot be surely concluded [3]. In recent times, innovative clinical trial designs using advanced statistical methods, simulation programs to emulate real-life trial designs and scenarios, and usage of real-world evidence (RWE) to assess and determine clinical endpoints for study design are some of the approaches proposed by regulators.

## Significance of the study

This study employs a review of various literature types encompassing review papers, concept papers, points of view, empirical researchers, policy guidelines, and expert reviews to explore the challenges faced by researchers in conducting rare disease trials in the EU as well as patient perspectives, and regulatory hurdles.

It aims to provide key insights by incorporating views and findings from various sources by adopting a fresh perspective considering the evolving landscape of rare disease policies and increasing focus on orphan drug research based on the latest scientific advancements. This study also aims to introduce actionable recommendations beyond theoretical practices and general solutions by incorporating suggestions based on technical advancements in health tracking, disease progression modeling using natural history data, and treatment outcome assessments and provides perspectives for conducting future research in this domain. It focuses on the specific regional nuances of the EU and attempts to identify strategies that will fit into the orphan drug development and regulatory framework of this region.

This study adopts an interdisciplinary approach to promote innovative trial designs led by improved policy decisions, simulation modeling and effective usage of registry and natural history data, novel endpoint and biomarker-based effectiveness assessments, adopting innovative drug development planning, and improving collaboration across the healthcare value chain.

## Methodology

### Study selection and data collection

This study was conducted following the Preferred Reporting Items for Systematic Reviews and Meta-Analyses (PRISMA) guidelines. Refer PRISMA Checklist for details. The criteria for the selection of relevant studies and documents and for performing the necessary assessment of relevant literature are provided below.

### Search strategy and selection criteria

Search was performed to retrieve relevant studies, policy documents, and online materials from PubMed, SCOPUS, BMJ, JAMA, Nature, Web of Science collection, Taylor and Francis Online, and internet search. Search terms were categorized into three parts:

- Population: “rare disease OR orphan drugs” AND (Europe OR European Union OR EU OR “multicountry”).
- Intervention: “clinical trial” OR “clinical research” OR “drug development”.
- Outcomes: “regulatory challenges” OR “regulatory hurdles” OR “innovative designs” OR “patient recruitment” OR “trial design” OR “methodology” OR “regulatory strategies” OR “real-world data” OR “natural history data”.

The indexing was performed using the following search query:

(“rare disease” OR “orphan drug “) AND (“Europe” OR “European Union” OR “EU” OR “multicountry”) AND ((“clinical trial” OR “clinical research” OR “drug development”) AND (((“regulatory challenge” OR “regulatory hurdle”) OR “regulatory strategy” OR (“innovative design” OR “trial design” OR “methodology”) OR (“patient recruitment” OR “patient engagement”) OR (“real world data” OR “real world evidence”) OR “natural history data”).

The data related to the clinical trial landscape were extracted from the International Clinical Trials Registry Platform (ICTRP) of the World Health Organization and the EU-related data were filtered based on the EU Clinical Trial registry.

We tailored data extraction to fit the study’s qualitative and narrative nature, systematically retrieving key meta-data like document details, publication dates, objectives, designs, policies, shortcomings, opportunities, and findings. Two independent reviewers ensured the accuracy of data by reviewing various aspects covered in the documents.

### Inclusion and exclusion criteria

The content considered for inclusion comprised studies that report on the regulatory and clinical challenges of conducting rare disease clinical trials in the EU and focused on regulatory and innovative strategies to address the same, studies including commentaries, reviews, and editorials that provide recommendations or solutions to overcome the challenges of rare disease clinical trials in the EU, studies that are published in English or have an English abstract available and studies that are published from 2005 onwards, to reflect the evolving regulatory and clinical landscape of rare disease research and trials in the EU. Also, articles and web publications in peer-reviewed journals or websites and from reputable organizations were considered for study.

Studies that do not focus on rare disease clinical trials, or only mention them as a secondary or minor aspect, studies that did not address the EU context, or only mention it as a secondary or minor aspect, Studies that provided a generic overview of these aspects and have methodological limitations and studies not directly addressing the specifics of the challenges and not providing any insight into innovative trials and clinical evaluation or planning were excluded from the study.

23 journal articles and 30 web publications (position papers, editorials, frameworks, guidelines, and details of stakeholder organizations in the EU rare disease landscape) were considered to conduct this study.

## Discussion

The research focus and findings of the 23 journal articles are provided in Table 1. Please refer below:

**Table 1:** Summary of research findings in the reviewed literature

Study	Research Focus	Aim	Key findings
Hilgers (2016)	Rare disease clinical trials	To review and improve the design and analysis methods for clinical trials in small rare disease populations.	The design and analysis methods for clinical trials in small rare disease populations have various challenges and there are limitations to applying traditional statistical methods to rare disease trials. Some alternative approaches such as adaptive designs, Bayesian methods, extrapolation, and meta-analysis, and more collaboration and harmonization among researchers, regulators, patients, and industry will facilitate the development of new therapies for rare diseases.
Kempf et al. (2018)	Clinical trials in rare disorders	To review the challenges and strategies of developing and conducting clinical trials in rare disorders.	Clinical trials in rare disorders face difficulties such as limited patient numbers, heterogeneity, lack of natural history data, and variability in endpoints. Different study designs and statistical methods can be used to increase the efficiency and validity of clinical trials in rare disorders. Regulatory agencies have shown flexibility and support for novel approaches to clinical trials in rare disorders. Collaboration and communication among stakeholders are essential for successful clinical trials in rare disorders.
O’Connor & Hemmings (2014)	Small populations issue in clinical trials	To review the challenges and approaches for clinical trials in rare diseases	The authors discuss various study designs and methods to increase the efficiency and utility of clinical trials in small populations. They also highlight the regulatory considerations and flexibilities, as well as the ongoing research initiatives in this area.
Day et al. (2018)	Small population clinical trials	To present recommendations for the design of trials involving small patient population	The authors discussed six topics: different study methods/designs, adequate safety data, multi-arm trial designs, decision analytic approaches, extrapolation, and patients’ engagement in study design. They suggested to consider alternative trial design options, combine safety data from different sources, support multi-arm trials via international networks, engage patients in trial design and therapy development, and seek input from multiple regulatory agencies
Mellerio (2022)	Clinical trials in rare diseases	To discuss the challenges of conducting clinical trials in the field of rare diseases and to highlight the specific considerations and potential pitfalls in the rare disease arena.	The article discusses the obstacles and challenges faced in both commercial trials and academically sponsored studies. These include questions around trial design, recruitment targets, mitigating dropout, and challenges of regulatory approval if the bar for efficacy and safety are met. The article further elaborates on the specific considerations and potential pitfalls in the rare disease arena for researchers, patients, pharma, and regulators.
Bell and Smith (2014)	Interventional clinical trials in rare diseases	To provide a comprehensive characterization of rare disease clinical trials registered in ClinicalTrials.gov, and compare against characteristics of trials in non-rare diseases.	Of the 24,088 trials categorized, 2,759 (11.5%) were classified as rare disease trials, and 21,329 (88.5%) were related to non-rare conditions. Despite the limitations of the database, it was found that rare disease trials differed from non-rare disease trials across all characteristics that were examined
Ndebele et al. (2014)	Multi-country clinical trials in resource-limited settings	To address the regulatory challenges associated with conducting multi-country clinical trials in resource-limited settings. The authors sought to ensure that the trials meet regulatory requirements in all countries in which the clinical trials will be conducted and also explore how national laws can hinder or expedite clinical trials and drug approval based on regulatory expectations.	A limiting factor to the efficient conduct of multi-country clinical trials is the regulatory environment in each collaborating country, with significant differences determined by various factors including the laws and the procedures used in each country. The long regulatory processes in resource-limited countries may hinder the efficient implementation of multisite clinical trials, delaying research important to the health of populations in these countries and costing millions of dollars a year.
McCormack et al. (2013)	Social and ethical issues in neuromuscular rare disease-related care and therapy development	To provide guidance in social and ethical issues related to clinical, diagnostic care, and novel therapies for hereditary neuromuscular rare diseases. The researchers sought to address the complexities added by the possibility of genetic, mutation-specific treatments. They also aimed to think through the implications of adopting a personalized medicine approach.	Drug trials in children engage with many ethical issues, from drug-related safety concerns to communication with patients and parents, and recruitment and informed consent procedures. This paper addresses the field of neuromuscular disorders where the possibility of genetic, mutation-specific treatments, has added new complexity. Not only must trial design address issues of equity of access, but researchers must also think through the implications of adopting a personalized medicine approach.
Baumfeld et al. (2019)	Hybrid Trial designs using real-world data	To explore how hybrid study designs that include features of randomized controlled trials (RCTs) and studies with real-world data (RWD) can combine the advantages of both. Researchers sought to generate real-world evidence (RWE) that is fit for regulatory purposes. The researchers also aimed to develop a hybrid trial methodology combining the best parts of traditional RCTs and observational study designs.	Some hybrid designs include randomization and use pragmatic outcomes; other designs use single-arm trial data supplemented with external controls. These approaches have already been successfully used in regulatory decisions, raising the possibility that studies using RWD could increasingly be used to augment or replace traditional RCTs for the demonstration of drug effectiveness in certain contexts. These changes come against a background of long reliance on RCTs for regulatory decision-making, which are labor-intensive, costly, and produce data that can have limited applicability in real-world clinical practice.
Mulberg et al. (2019)	Regulatory strategies to address challenges in rare diseases therapy development	To improve rare disease clinical development strategies under current global regulatory statutes. This was achieved by creating a position paper based on a meeting with representatives from the FDA, the biopharmaceutical industry, and not-for-profit agencies.	This study identified several strategies to minimize the limitations associated with low patient numbers in rare diseases, including the use of natural history to generate historical control data in comparisons, and simulations, and identifying inclusion/exclusion criteria and appropriate endpoints. Novel approaches to clinical trial design were discussed to minimize patient exposure to placebo and to reduce the number of patients and clinical trials needed for providing substantial evidence. Novel statistical analysis approaches were also discussed to address the inherent challenges of a small patient population.
Manolis and Pons (2009)	Model-based paediatric medicinal development	To identify the regulatory framework for the use of modeling and simulation (M&S) in paediatric medicinal development and to make proposals for model-based paediatric medicinal development.	As per pediatric European Union (EU) regulation and the consequent demand for pediatric studies on one hand and the ethical need for minimizing the burden of studies in children, on the other hand, necessitate optimal techniques in the assessment of safety/efficacy and use of drugs in children. Modeling and simulation (M&S) is one way to circumvent some difficulties in developing medicinal products in children. M&S allows the quantitative use of sparse sampling, characterization, and prediction of pharmacokinetics/pharmacodynamics (PK/PD), extrapolation from adults to children, interpolation between pediatric age subsets, optimal use of scientific literature, and in vitro/preclinical data.
Cox (2018)	Efficacy endpoints in rare disease clinical trials	To address the challenge of demonstrating a clinically meaningful and statistically significant response to treatment in rare disease clinical trials. To discuss the intricacies of selecting the most appropriate and sensitive efficacy endpoints for a treatment trial.	The paper discusses the importance of selecting the most appropriate and sensitive efficacy endpoints for rare disease clinical trials. It emphasizes that this selection process is part art and part science. The study highlights that for rare diseases, regulatory approval requires demonstration of clinical benefit, defined as how a patient feels, functions, or survives, in at least one adequate and well-controlled pivotal study conducted according to Good Clinical Practice. In some cases, full regulatory approval can occur using a validated surrogate biomarker, while accelerated, or provisional, approval can occur using a biomarker that is likely to predict clinical benefit.
Crow et al. (2018)	Rare disease clinical trial hindrances and points of action	To identify systematic problems in the set-up of international, multi-center clinical trials using the FOR-DMD study as an example. The full timeline of the FOR-DMD study, from funding approval to site activation, was collated and reviewed.	The study found that time from the first contact to site activation across countries ranged from 6 to 24 months. Reasons for delay were universal (sponsor agreement, drug procurement, budgetary constraints), country-specific (complexity and diversity of regulatory processes, indemnity requirements), and site-specific (contracting and approvals). The main identified obstacles included [1] issues related to drug supply [2], NIH requirements regarding contracting with non-US sites [3], differing regulatory requirements in the five participating countries [4], lack of national harmonization with contracting and the requirement to negotiate terms and contract individually with each site and [5] diversity of communication.
Kakkis et al. (2015)	Development of rare disease drugs through specific regulatory protocol	To propose a scientific framework for assessing biomarker endpoints to enhance the development of novel therapeutics for rare and devastating diseases.	The paper provides specific recommendations including establishing regulatory rationale for increased Accelerated approval access in rare disease programs, implementing a Biomarker Qualification Request Process to provide the opportunity for an early determination of biomarker acceptance, and a proposed scientific framework for qualifying biomarkers as primary endpoints1. The paper also highlights case studies of successful examples that have incorporated biomarker endpoints into FDA approvals for rare disease therapies.
Maca et al. (2006)	Adaptive and Seamless designs for Phase2/3 clinical trials	To introduce the concept of adaptive designs and describe the current statistical methodologies that relate to adaptive seamless designs. The researchers also aimed to describe the decision process involved with seamless designs and present some illustrative examples.	Adaptive seamless designs have been considered as one possible way to shorten the time and patient exposure necessary to discover, develop, and demonstrate the benefits of a new drug. The researchers introduced the concept of adaptive designs and described the current statistical methodologies that relate to adaptive seamless designs. They also described the decision process involved with seamless designs and presented some illustrative examples.
Hilgers, et al. (2018)	Design and analysis of small population clinical trials.	Assessing the impact of IDeAl project to develop new statistical design and analysis methodologies for clinical trials in small population groups.	The IDeAl project provided 33 practical recommendations for researchers, helping them design and analyze efficient clinical trials in rare diseases with a limited number of patients available. The project’s findings are expected to refine the statistical methodology for small-population clinical trials from various perspectives.
Moore et al. (2022)	Improving rare disease clinical studies through decentralized trials.	To explore the role of decentralized trial designs in improving rare disease studies. The researchers sought to understand how these designs could become a standard approach, especially in the wake of changes necessitated by the COVID-19 pandemic.	The study found that decentralized clinical trial (DCT) designs have been developed in some rare disease trials and changes necessitated by the COVID-19 pandemic present an opportunity for them to become a standard approach. DCT approaches have been shown to be more resilient to changes in enrolment and attrition during COVID-19 than traditional designs and offer benefits in terms of patient burden, convenience, inclusion, and data quality. Digital tools such as wearable devices and electronic clinical outcome assessments may also provide more convenient and environmentally valid measures of how a condition affects the life of an individual in their regular environment. However, challenges exist, such as technical support, the digital divide, ensuring high-quality data, and delivering safe trials.
Ghadessi et al. (2023)	Decentralized clinical trials for rare diseases	To discuss the planning and conduct of Decentralized Clinical Trials (DCTs), which can increase the quality of trials with a specific focus on rare diseases.	Traditional clinical trials require tests and procedures that are administered in centralized clinical research sites, which are beyond the standard of care that patients receive for their rare and chronic diseases. The limited number of rare disease patients scattered around the world makes it particularly challenging to recruit participants and conduct these traditional clinical trials. Participating in clinical research can be burdensome, especially for children, the elderly, physically and cognitively impaired individuals who require transportation and caregiver assistance, or patients who live in remote locations or cannot afford transportation.
Burns et al. (2022)	Real-world evidence for regulatory decision-making.	To assess the global regulatory environment with regard to real-world evidence (RWE) based on regional availability of the following 3 key regulatory elements: (1) RWE regulatory framework (2), data quality and standards guidance, and (3) study methods guidance.	The study reviews the available frameworks and existing guidance from across the globe and discusses the observed gaps and opportunities for further development and harmonization. It encourages cross-country collaborations to further shape and align RWE policies and help establish frameworks in countries without current policies to create efficiencies when considering RWE to support regulatory decision-making globally.
Mitra et al. (2023)	Review of rare oncology therapeutics approvals in USA.	To understand the regulatory landscape as it relates to the application of clinical pharmacology principles in rare oncology product development and to understand the expected clinical pharmacology studies and knowledge base in such approvals and the role of Model-informed drug development (MIDD).	The findings highlighted how clinical pharmacology contributed to the evidence of effectiveness, dose optimization, and elucidation of intrinsic and extrinsic factors affecting drug behavior. Clinical pharmacology studies were often integrated with modeling in many of the NDAs/BLAs. The approaches undertaken for oncology drug approvals can provide future directions for conducting clinical studies for drugs for other rare diseases in different therapeutic areas.
Lasonos and O’Quigley (2021)	Randomized Phase 1 clinical trials for oncology studies.	To broaden the objectives of Phase 1 trials in oncology to address multiple objectives under the heading of early-phase trials and, if possible, obtaining reliable evidence regarding clinical activity to lead to drug approvals via the Accelerated Approval approach or Breakthrough Therapy designation.	The article outlines objectives and design considerations that need to be adhered to in order to respect ethical and scientific principles required for research in human subjects in early-phase clinical trials. The authors articulate the role of randomization in early-phase trials where the goal is to go beyond the simple comparison of different drugs. They provided examples of relevant, time-clinical questions where randomization can help avoid potential biases in data collection and their interpretation.
Rollet et al. (2013)	Sustainable rare diseases businesses based on economic and pricing dynamics.	To address misconceptions around pricing dynamics and rare-diseases business models, and to ensure the successful continuation of a dynamic Orphan Medicinal Products (OMPs) R&D within rare-diseases public health policy.	The authors argue that misconceptions about the pricing of rare diseases drugs reflect a poor appreciation of the R&D model and the affordability and value of OMPs. They suggest that the potential financial returns of small medium-sized rare diseases companies focusing on high-priced drugs and shared burden of manufacturing for scale-up in regards to drug development.
Kooiker (2019)	EU regulatory initiatives overview	To present a broad overview of existing legislation, programs, and incentives in the EU that allow for accelerated access to orphan drugs and high medical need products, as well as give some historical context and how these programs are interconnected.	The authors conducted an explorative review of publicly available sources of regulatory and orphan drug policies as well as information on accelerated assessment programs in the EU. They identified and looked into various programs and initiatives, either national or EU-wide. Hurdles in regulatory science remain present, especially for rare diseases, but fundamental research as done in FP7 could lead to more efficient orphan drug development. Barriers in the field of HTA, pricing, and reimbursement still need to be addressed to enhance patient access.

The studies of rare disease trials have proposed key conceptual frameworks and have also provided empirical insights into the design, conduct, evaluation, and outcome of these trials. A baseline synthesisation of key findings from these studies provides a core observation that the design and conduct of clinical trials can be assumed to be centered around three themes: Disease characteristics, Trial Design and Methodology, and Clinical Outcome Assessment. The core concept of clinical research plans begins with understanding specific rare diseases, their heterogeneity, and natural history, which inform Trial Design and Methodology, including study objectives, endpoints, and ethical considerations. Clinical outcomes and impacts, such as efficacy, safety, patient-reported outcomes, and cost-effectiveness, are influenced by the chosen methodology and may vary across different ethnic groups and even within patients at various stages of the disease or encountering specific molecular subsets of the disease. Relationships between these themes highlight the iterative nature of clinical research and especially in the case of rare diseases these iterations may not be sequential but in most cases need to be concurrent and flexible to ensure maximum and diverse patient coverage. Incorporating the themes in a common regulatory and funding framework across the EU becomes challenging due to huge ethnic, geographical, financial, infrastructural, and cultural diversity. Stakeholder perspectives, comparative analysis, ethical considerations, and future directions add depth to ongoing drug development initiatives. This approach warrants the inclusion of diverse viewpoints, ethical awareness, ongoing refinement of research practices, and continuous innovation in the design and conduct of trials to address the unique challenges and opportunities in rare disease clinical trials within the EU.

### Landscape of orphan drug clinical trials in the EU:

#### Clinical trials conducted based on therapeutic areas in the EU

**Fig. 1:**
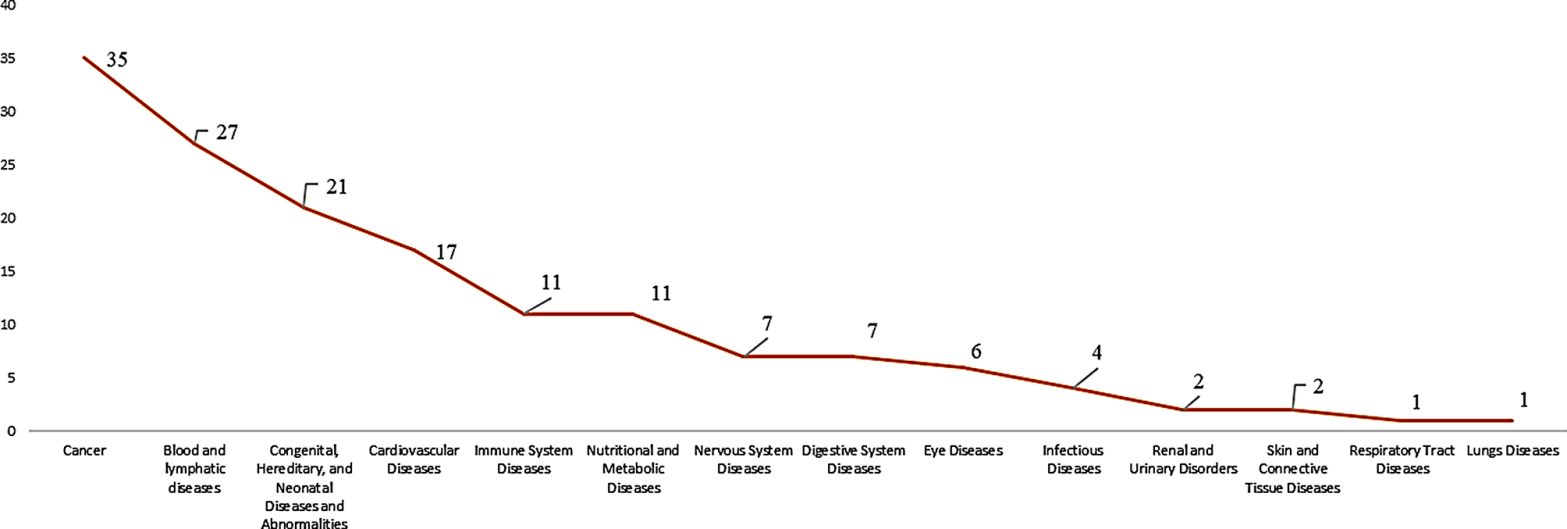
Orphan drug clinical trials based on therapeutic area (2007–2022) (EU Clinical Trial Registry). *Source* International Clinical Trials Registry Platform (ICTRP) [9]

Between 2007 and 2022, 152 clinical trials were conducted on orphan drugs in the EU, as recorded in the EU Clinical Trial Registry. Refer to Fig. 1. 23% of the trials were on cancer-related therapies, followed by blood disorders with an 18% share of the trials and congenital abnormalities with a 14% share of the trials. The rest of the trials were related to other therapeutic areas, such as cardiovascular diseases, immune system diseases, and nervous disorders.

### Yearly spread of clinical trials of orphan drugs in the EU

**Fig. 2:**
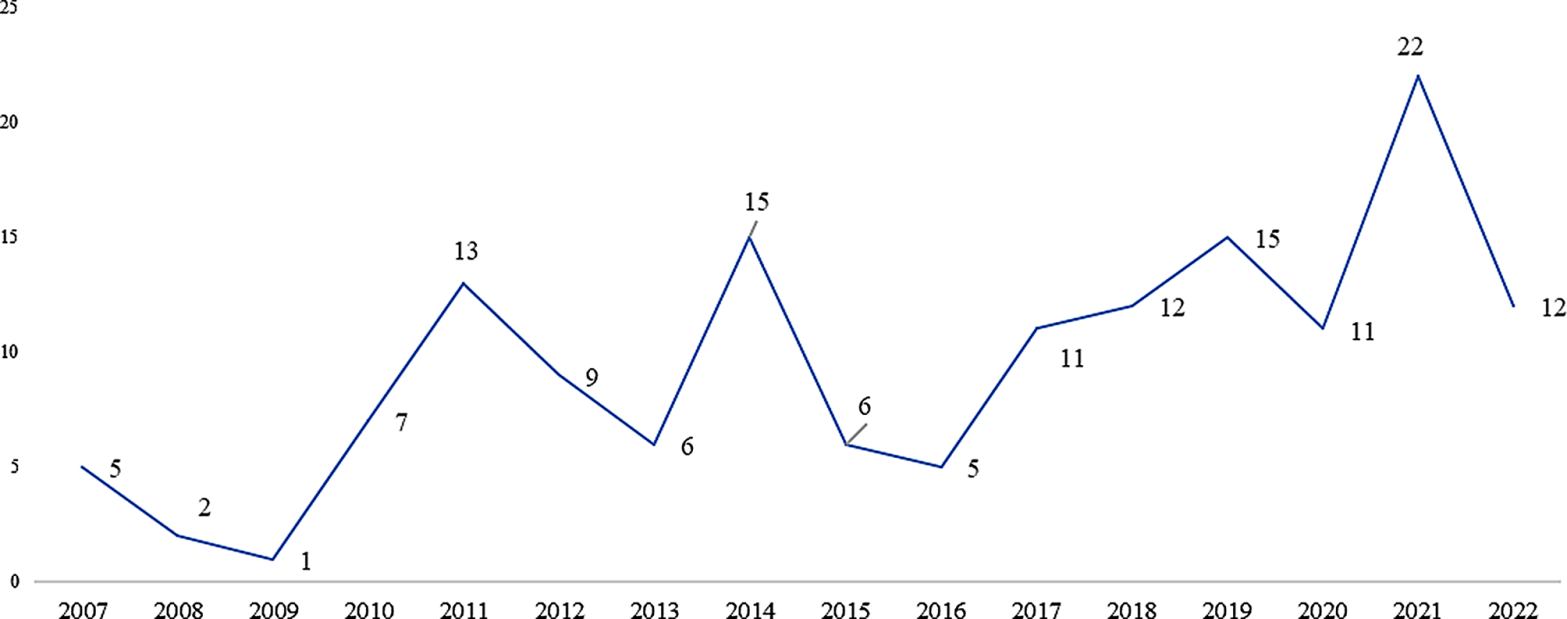
Yearly orphan drug clinical trials in EU (2007–2022). *Source* International Clinical Trials Registry Platform (ICTRP) [9]

Between 2007 and 2022, clinical trials on orphan drugs have seen both an increase and a decrease on a year-on-year basis, with a decrease observed during the intermittent period mainly in 2015 and 2016. Refer to Fig. 2. After that, the trend has been upward again, with 22 trials being conducted in 2021, which is the highest in the period under observation. The number again decreased to 12 in 2022. There is an average increase of 51% in the clinical trial volume from 2007 to 2022.

### Clinical trial volume of orphan drugs based on product types in the EU

**Fig. 3:**
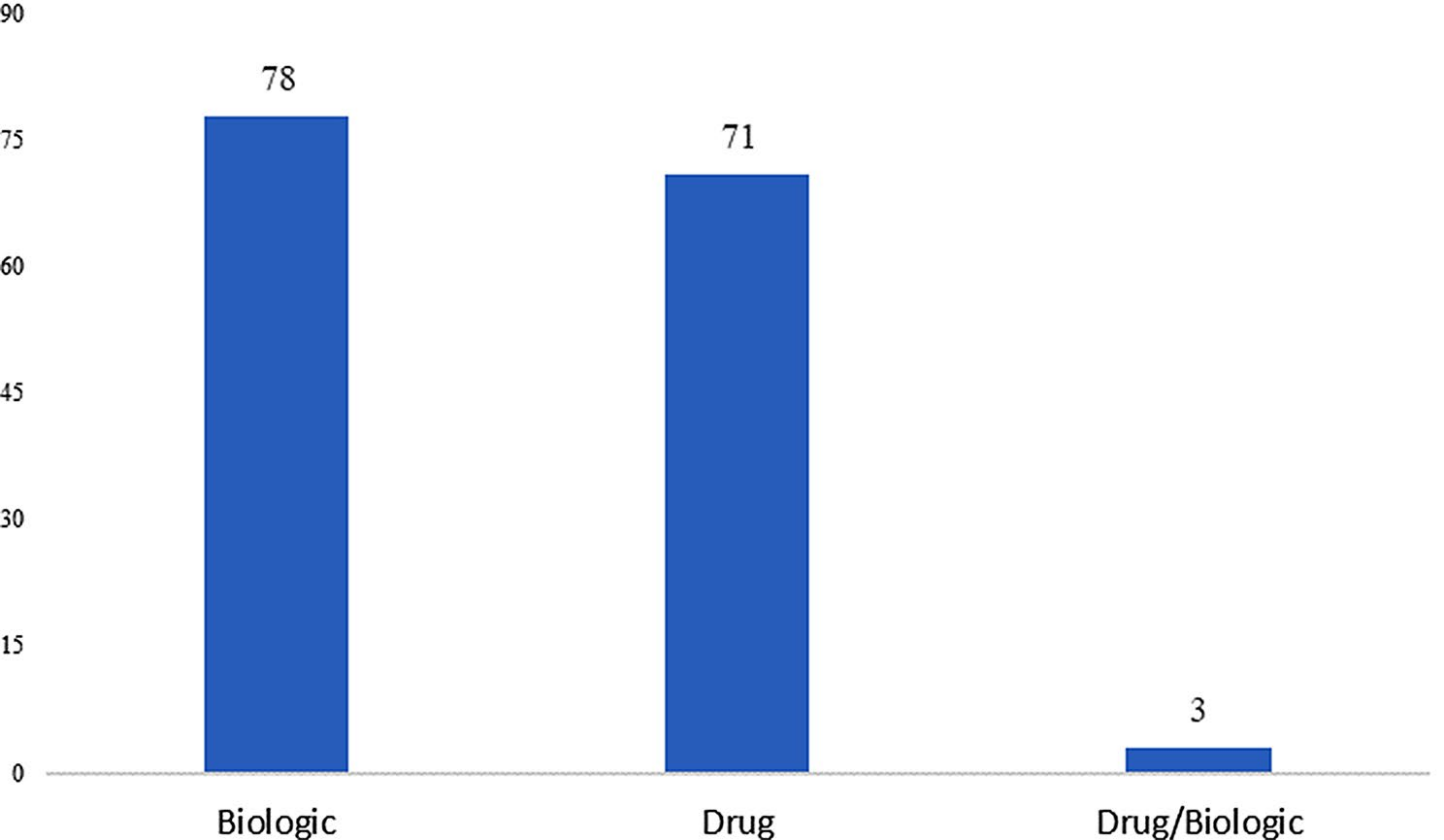
Orphan drug trial volume based on product type in EU (2007–2022). *Source* International Clinical Trials Registry Platform (ICTRP) [9]

Between 2007 and 2022, biological products underwent 78 trials compared to 71 trials for synthetic drugs. Refer to Fig. 3. This might be an indication that investments made by the EU in the field of biotechnology have created a favorable environment for sponsors for biologic development. The EU market provided potential commercial opportunities due to less competition in the field of biologics. Biologics may also be favored due to their targeted mechanism of action, high specificity and efficacy, and ability to modulate complex biological processes underlying the specific disease being treated. These factors make biologics more suitable therapies than synthetic drugs for diseases belonging to certain therapeutic areas.

### Top 10 companies conducting orphan drug clinical trials in the EU

**Fig. 4:**
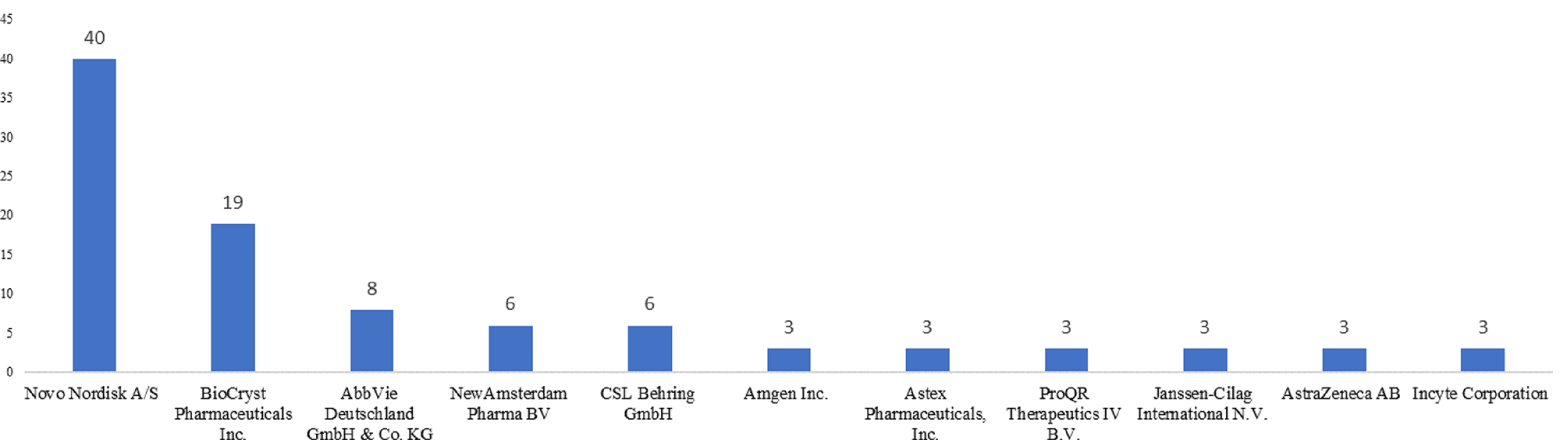
Top 10 companies in terms of orphan drug trials in EU (2007–2022). *Source* International Clinical Trials Registry Platform (ICTRP) [9]

Between 2007 and 2022, Novo Nordisk conducted the maximum number of clinical trials focusing on congenital, hereditary, and neonatal diseases and abnormalities, nutritional and metabolic diseases, digestive system diseases, cardiovascular diseases, and blood and nervous disorders. It was followed by BioCryst Pharmaceuticals Inc., which conducted 19 trials focusing on blood disorders and immune system diseases. The top 10 companies contributed to 64% of all orphan drug clinical trials in the EU between 2007 and 2022. Refer to Fig. 4.

**Fig. 5:**
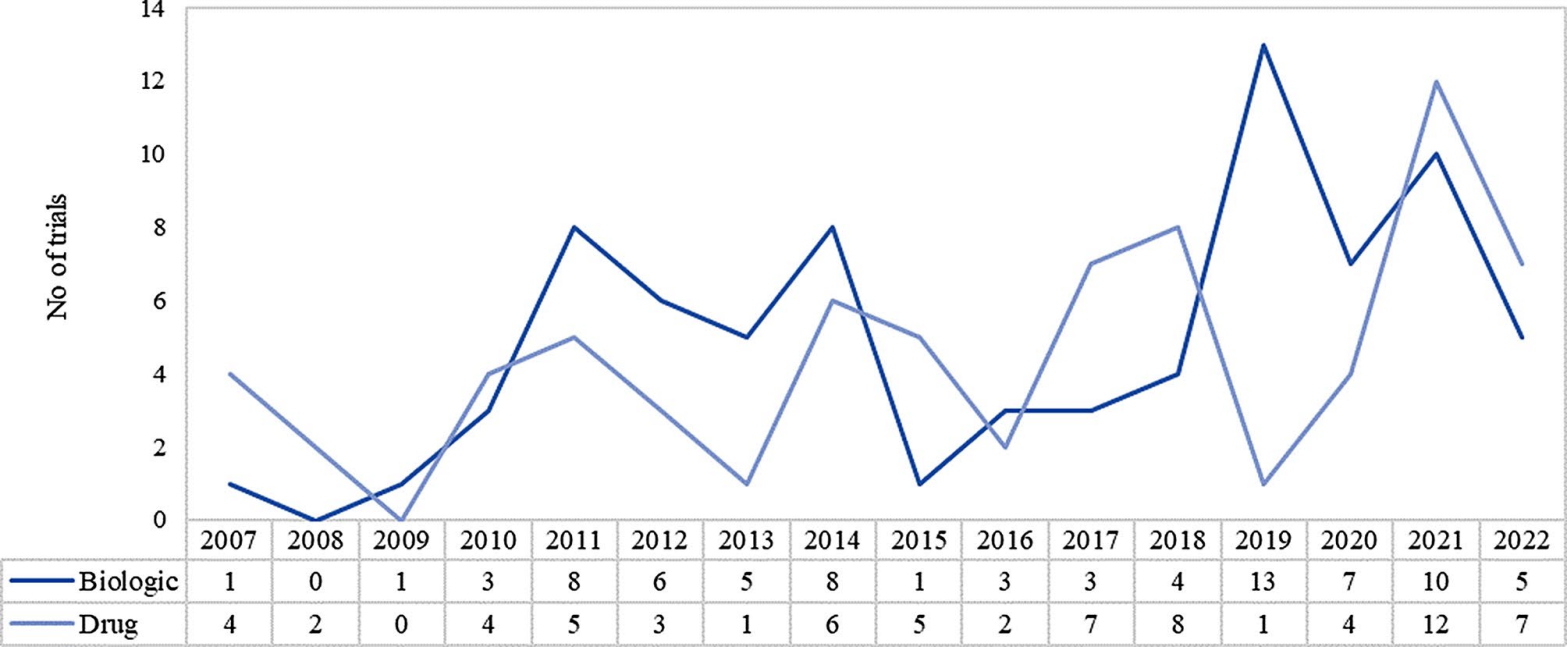
Yearly trend of clinical trials of orphan drugs based on product types in EU (2007–2022). *Source* International Clinical Trials Registry Platform (ICTRP) [9]

Clinical trials on biologics have seen an increase between 2019 and 2022 primarily due to an increased focus on biologics development for rare disease therapies. This is also backed by the growing knowledge of genetic markers of specific diseases that help in the development of targeted therapies for patients. It was observed that 45% of all biologic therapeutic trials between 2007 and 2022, were carried out between 2019 and 2022. Refer to Fig. 5.

### Inferences from landscape data

Companies have been showing an increased interest in orphan drug development in the EU, primarily supported by the EMA’s orphan drug development incentives such as ten years of market exclusivity for orphan-designated products, centralized and accelerated review of marketing authorization applications for orphan products, conditional marketing authorization of certain drug types, compassionate access under exceptional circumstances for patients with high morbidity, application and regulatory fee waivers and EC research frameworks and grants for orphan drug innovation and rare disease natural history studies. Most of the major pharmaceutical companies have developed and conducted trials on a considerable number of orphan drugs across different therapeutic areas. This, supported by collaborative programs by European Reference Networks (ERNs), the European Joint Program on Rare Diseases (EJP-RD) [10, 11], and the International Rare Diseases Research Consortium (IRDiRC) [12] has played a major role in creating a translational research environment for orphan drug development and innovative trial design. Many small and medium enterprises (SMEs) working on rare disease innovation in the biotechnology and pharmaceutical domain are supported by the European Confederation of Pharmaceutical Entrepreneurs (EUCOPE) in terms of financial incentives, development support, and scientific advisory. EUCOPE also acts as a bridge for advancing translational research. Qualified SMEs receive additional incentives from the EMA like a 100% fee reduction for administrative and procedural assistance, pre-authorization inspection, initial marketing authorization application and post-authorization applications, and annual fee, specified in Council Regulation (EC) No 297/9554, in the first year from granting of a marketing authorization. The development of unified patient databases through the European Rare Disease Registry Infrastructure (ERDRI) and Patient-Reported Outcome and Quality of Life Instruments Database (PROQOLID) [13] database by Mapi Research Trust has provided valuable information on Real World Data from patient treatment outcomes, valuable biomarkers, diagnostic reports, and observer as well patient experience feedback [14]. Although there have been definitive steps taken at the EC level, regulatory agency level, and by research consortiums and patient organizations such as the European Organization for Rare Diseases (EURORDIS) [15], the pace of innovation and marketing approval of drugs is not as expected, as is evident from the overall analysis presented in the landscape section Apart from the practical challenges that are discussed in a later section, ineffective utilization of regulatory incentives and the requirement of high capital investment have been factors behind a slower pace of therapeutic development. Along with the key requirements of addressing clinical trial issues, it is equally important to implement an alternative approach to drug development planning to increase the participation of players in orphan drug clinical research and development.

### Key aspects of rare disease trial designs

#### Practical challenges in conducting orphan drug clinical trials in the European Union

Clinical trials, being one of the most significant stages in the drug development lifecycle face various hurdles, especially in a multi-country setup like the EU. It includes finding eligible patients and overcoming awareness issues. International trials introduce further complexities like consensus on diagnosis and cultural considerations [4] Disease heterogeneity poses further challenges making progression tracking difficult, and varied manifestations across different patient groups create challenges in diagnosis, treatment, and understanding effectiveness [5]. Genetic factors, variability in disease frequency among patient groups [3], lack of knowledge about disease progression [7], and co-existing illnesses complicate the determination of inclusion/exclusion criteria and the tenure of trials [8, 16]. In many cases, RCTs fail to deliver the desired trial outcomes in normal settings. Limited knowledge about appropriate endpoints complicates protocol design, hindering regulatory approval. Small patient populations and data variability exacerbate challenges in demonstrating drug effectiveness and safety, compounded by the absence of standardized clinical trial templates for most rare [6, 17]. Balancing risks and benefits, respecting autonomy, and ensuring equal access are crucial issues. The limited treatment options make patients more likely to accept risks, raising concerns about informed consent which becomes a critical ethical issue [17]. Evolving regulations with stricter requirements and limited expert resources can delay research [18]. Regulatory processes and differing policies across countries add complexity to the design and conduct of rare disease trials in the EU [19]. Regulators must rely on literature review information and empirical data to a large extent when making decisions. Data extrapolation is followed for diseases with existing data, while for diseases with little prior data, several iterations need to be performed across different patient population samples resulting in cost overruns or high expenditure for the sponsors. A considerable patient population of rare disease patients is children, posing challenges in recruitment, retention, and management due to factors like developmental, emotional, and family dynamics. The varying clinical research approaches for pediatric and adult use intensify complexities in recruitment criteria, consent, and regulatory acceptance of study outcomes, amplifying oversight of ethical aspects in conducting trials [20]. In terms of costs, rare disease clinical trials incur high costs due to challenges such as the geographical spread of patients, complex trial protocols, and expensive manufacturing overheads to meet regulatory standards. Specialized trial designs may be necessary to recruit the required number of patients, further escalating expenses. Additionally, data collection and analysis using sophisticated statistical models, drive up the overall cost of drug development. Hence, this factor becomes one of the key components in drug pricing, treatment availability, and reimbursement as well. Countries like Germany, with a strong economy and financial budget, have the privilege to establish initial prices of innovative therapies making it one of the initial markets for product launch. This results in a price that may be attractive to the sponsor company but may pose a significant financial burden on countries with less financial resources and undeveloped reimbursement schemes. The downside of high pricing needs to be considered as well. Any kind of failure in price negotiations for high-cost therapies with the national Governments results in the withdrawal of the therapeutic product from the entire EU market due to a lack of reference and attractive pricing for the company. An example of this was the withdrawal of Zynteglo, for the treatment of severe Beta thalassemia and of Skysona, for the treatment of Cerebral adrenoleukodystrophy by Bluebird Bio. Pricing remains a critical issue in rare disease drug development and treatment availability [21]. One approach to mitigate the cost issue, is to implement specialized National Action Plans for rare diseases as per recommendations of the European Council. From an economic point of view, it seems difficult to implement uniform research plans, funding mechanisms, and reimbursement schemes across all member states. To address this, the European Committee of Experts on Rare Diseases (EUCERD) came out with EUROPLAN indicators to monitor the progress of plans in individual member states. These indicators can help track the progress of the National Action Plans. Stronger economies can provide financial and administrative expertise and financial grants through the E-Rare (ERA-NET) research program on rare diseases. An increased public-private partnership should be encouraged to enhance the expertise in conducting clinical research through a multi-stakeholder collaborative approach. This will provide an initial booster to the weaker economies to streamline their action plans and frame appropriate funding and reimbursement mechanisms for rare disease drug development and treatment.

#### Technical aspects of clinical trial design for rare diseases

RCTs have been the globally accepted and most reliable clinical trial design among clinical researchers, investigators, and regulatory agencies for demonstrating the effectiveness of a drug. However, conducting multiple RCTs can be time-consuming, and expensive, and may not fully reflect real-world clinical settings. As a result, there is a growing interest in finding innovative approaches to enhance the efficiency of clinical research [22]. RCTs are designed with standardized and comprehensive outcome measurements to determine the safety of the drug under trial. RCTs generate substantial evidence to determine the effectiveness of the drug by incorporating bias-reduction techniques to reduce errors in observations [22]. 

Randomization helps to differentiate between treatment outcomes as well as the variability of outcomes within a group. However, despite the multiple benefits and robustness of the design of RCTs, the validity of these cannot be always confirmed in orphan drug scenarios due to the small patient population. This is due to sampling techniques being unable to provide sufficient observation points. The design of orphan drug trials requires multicentre coordination across different locations in the world [8]. This requires the design and analysis of innovative trials through appropriate randomization procedures for a smaller population of patients. As the patient population varies across diseases as well as across demography or geography, there are always differing amounts of bias. Hence, it is imperative that no unique procedure or randomization technique is applicable, and it should be supported by proper design methodology, statistical tests, and analytical methods [3]. 

#### Rare disease clinical trial design considerations

Randomized trials are highly dependent on patient registry information to identify specific representative populations. Periodic reference to the updated registries can help sponsors improve study design and determine study cohorts to conduct case-control and observational studies. This helps in bias reduction but there are also certain challenges in terms of rare disease trial design. Existing registries may not have adequate information and validation of the correctness becomes a challenge due to highly fragmented content, lack of expertise, and data sharing or privacy controls. From a disease perspective, the progression of rare diseases is highly heterogeneous and varies between demographics and geographical dispersions. Genetic markers within the same geographical area can vary between communities with few communities highly susceptible to the disease due to cultural or lifestyle practices. In this scenario, it is difficult to determine the proper natural history and clinical endpoint based on diagnostic and treatment outcomes [3]. 

#### Analytical and statistical considerations for clinical trial designs

While statistical sampling and analytical methodologies are proven to be highly effective in identifying patient subsets and trial effectiveness outcomes for RCTs for nonorphan drug trials, the same methodologies may not prove effective in the case of rare disease trial designs. It is important to identify efficient design and analysis techniques. While it is recommended to adopt well-defined, widely used, and regulatory-approved techniques, evaluations need to be performed and continuous monitoring is required to establish method validity and adaptability to various trial designs for rare diseases [3]. In studies of rare diseases, small sample sizes can severely restrict design options, hinder the usage of standard statistical models as mandated by regulators, and make replication of study models difficult. To assess the efficacy and safety of potential treatments, it is imperative to explore novel and innovative statistical designs [23]. It is also important to identify an appropriate observation population and study cohort to ensure that bias is properly addressed by using computer simulation models and advanced statistical techniques. While computer simulation methodologies can address the population issue to a considerable extent by identifying patient cohorts for randomized studies; it is also important to systematically study the interactions between treatment and disease phenotypes to prepare targeted trials to understand drug-patient interactions under personalized settings and individual requirements. Statistical algorithmic models can prove helpful in this regard. Digital endpoints generated from sensors installed in wearables can provide valuable real-time data that can be fed to computer simulation models to identify appropriate biomarkers and generate clinically relevant endpoints or surrogate endpoints [24]. However, there is a challenge, as identifying relevant and meaningful data from multiple observations in the form of digital data is time-consuming and requires multiple iterations.

### Recommendations for improving the clinical trial design of rare diseases

#### Effective usage of natural history and disease registry data for trial design

The strategic utilization of natural history and disease registry data plays a pivotal role in the design of clinical trials for rare diseases. This data serves as a roadmap, providing valuable insights into the complex and unique progression trajectories of these diseases, which is an essential component in assessing the impact of new therapeutic interventions [25]. It is important to harness the crucial information around these studies to derive quantifiable biomarkers to effectively chart the therapeutic roadmap, design the trial along with devising a robust drug development plan to augment the possibilities of regulatory approval supported by appropriate safety and efficacy data. Studies have been conducted on Duchenne Muscular Dystrophy based on natural history data for identification of relevant biomarkers based on progression and severity and these data provided valuable data for regulatory assessment [26, 27]. It is to be noted that the landscape of natural history studies on rare diseases faces multiple challenges, including a limited pool of participants, issues with data quality, and the existence of data silos [28]. However, innovative solutions to design simulation models utilizing machine learning, are emerging to address these obstacles, fostering an environment of collaboration among stakeholders and promoting shared learning to enhance knowledge and expedite orphan drug discovery. Properly designed and maintained non-proprietary patient and disease registries based on real-world data, clinician and patient-reported outcomes and natural history study databases are cornerstones in this endeavor, offering a wealth of information about rare diseases [23]. These resources enable the design of robust clinical trials equipped with outcome measures that are both relevant and clinically meaningful. By harnessing this data, researchers can significantly enhance the design of clinical trials for rare diseases. This will lead to the development of more effective therapies in the EU, enable informed regulatory approvals, and pave the way for improved patient outcomes, marking a significant stride in developing and implementing a robust patient-centered approach toward orphan drug development.

#### Adoption of innovative clinical trial design

In clinical trial designs for orphan drugs, the process of obtaining approvals should take into account the outcome variations and underlying variability in disease manifestations. Due to the lack of a sufficient population, Phase 3 studies for orphan drug approvals tend to include a smaller number of patients, do not have placebo controls in many cases, and employ nonrandomized and unblinded trial designs, such as a single-arm design and surrogate endpoints, for assessing efficacy [3]. This requires proper planning, collaboration, and timeliness. As in most cases, rare disease trials involve multiple sites, and consensus is required between researchers and regulatory agencies in terms of the definition and classification of the disease under trial, identification of appropriate biomarkers and endpoints, and assessment of outcomes on commonly accepted standards. Under these circumstances, innovative trial designs such as basket trials and umbrella trials can help to address these issues. Basket trials enable researchers to investigate certain disease types with specific genetic biomarkers under a common trial design and protocol. Using this approach, researchers test the efficacy of targeted therapies by grouping patients based on molecular characteristics of the disease and provide valuable insights into the potential benefits of a treatment in rare diseases. These trials use a common targeted intervention. On the other hand, umbrella trials involve enrolling patients with the same disease but with different molecular or genetic subtypes. This design allows researchers to test multiple targeted therapies simultaneously within the same disease population, including rare diseases. Umbrella trials help identify which subgroups of patients may benefit from specific treatments, leading to more personalized and effective interventions. These trials use multiple targeted interventions. Both of these trials can also be designed using control groups through randomization, thus ensuring bias reduction and effective assessment of clinical outcomes. These trials have certain advantages, such as sharing the same control group to improve efficiency, reducing the likelihood of patients receiving a placebo, allowing comparisons between active substances and pooling of data from active treatments, and sharing of resources, thus leading to reduced trial costs and more efficient use of trial logistics [16]. In a rare disease setting, these trials may face some challenges around sponsor coordination in terms of multiple treatment trials, complex study design, competing and conflicting interests among stakeholders, operational challenges when international centers and multiple sites are involved, and implementing and following a common protocol. In this scenario, the ERICA, ERNs, patient advocacy groups, and centers of excellence can play an active role in identifying suitable patient populations and can act as co-ordinators or collaborators for designing, funding, and assisting in conducting trials through disease expertise, data sharing, execution management and assessing the outcomes of trials in multicenter settings [16]. 

#### Adaptive trial design for rare disease trials

Adaptive trial designs can offer flexibility and increase the efficacy of rare disease trials. Adaptive design trials encompass modified randomization procedures, the addition or discontinuation of treatment arms or doses, sample size adjustments based on interim results, adaptive patient population enrichment, and the incorporation of prespecified rules for efficacy. These designs in exploratory settings allow for the evaluation of various doses, regimens, and populations, focusing on the most favorable observations that will ensure promising results. They increase flexibility and acceptability and maximize the trial’s potential based on gathered data. Prespecified modifications maintain validity and integrity while adjusting elements of the study design [29]. The statistical approach of these trial designs enables modifications of study elements for minimizing errors, careful planning, and ensuring trial ethics and integrity [8]. While designing these trials, it is important to address and mitigate challenges around operational logistics, feasibility, and access to technical expertise. These are crucial considerations in designing clinical trials and special attention should be given to rare disease trials. Adequate study design expertise is necessary to ensure appropriate planning, for which experienced clinical researchers, statisticians, and healthcare professionals who are well-versed in rare diseases and orphan drug trial design, execution, and outcome assessment should be identified. Additionally, maintaining data and trial integrity becomes essential for post-interim analyses. This requires data storage and analytics planning. Addressing concerns related to bias in estimated treatment effects further strengthens the integrity of the trial, and outcomes and observations become more acceptable to regulators [8]. 

#### Clinical trials through inferentially seamless adaptive designs

In the adaptive seamless design, trials are merged, and analyses are seamlessly integrated by including data from patients enrolled both before and after the adaptation in the final analysis [30]. Adaptive seamless designs, particularly in the context of rare diseases, offer an appealing approach when traditional group sequential designs for assessing efficacy or futility may not be feasible due to limited sample sizes [31]. These designs integrate a Phase 2 study, which focuses on treatment selection, with a Phase 3 study for confirmatory testing. This integration allows for treatment selection and the re-evaluation of sample size at a predefined interim analysis [3]. The use of adaptive seamless designs in rare disease clinical trials offers several benefits. It maximizes patient data utilization, leading to stronger conclusions, while reducing the number of patients and saving time and costs in Phase 3. It improves target dose and participant selection, explores covariates between Phase 2 endpoints and Phase 3 outcomes, and provides valuable information on treatment effects and safety by following patients from terminated treatment groups. Additionally, it allows for treatment modifications, enhancing the chances of patients receiving safe and effective treatments [32]. Challenges arise when working with these designs, including the time required for their design and the need for appropriate analyses to account for potential bias in treatment effect estimates due to data combinations at different study phases [16]. 

#### Decentralized clinical trial design for rare diseases

Decentralized methods involve conducting assessments in alternative locations such as participants’ homes, local clinics, or digital platforms on mobile devices or computers and not at centralized medical facilities. Decentralized clinical trial (DCT) approaches that incorporate physical and virtual consultations, along with online access to medicines or providing drugs through local clinicians or pharmacists, can bring substantial benefits by reducing the burden on patients and their families [33]. DCTs do not completely remove the physical interactions between clinicians and patients. It leverages digital health technologies (DHTs) such as medical devices and wearables. for electronic collection and usage of reliable diagnostic and clinical data, clinical outcome assessments (COAs), Patient and Observer Reported Outcomes, and clinical health records. These enable clinicians to determine exploratory patient-relevant endpoints, enable targeted patient recruitment, and help in collecting and updating data registries for secondary usage in future trials as reference points. This integrated approach will help clinical trial design be more focused on the outcomes. DCTs have their setup challenges in terms of technology adoption and usage, data privacy and technology literacy concerns, and site readiness with appropriate infrastructure, trained personnel, and logistical support. Keeping in mind the needs of patients, industry and clinical stakeholders need to upgrade technical aspects and knowledge of data collection and analysis [34]. Addressing data privacy concerns while sharing data across multiple trial sites as per domestic and international regulations will ensure confidentiality and appropriate usage of data. If the challenges are properly addressed, DCTs can play a crucial role in terms of increased adoption in conducting rare disease clinical trials and acceptance across regulators during decision-making.

#### Usage of external controls in designing effective rare disease trials

As rare disease clinical trials are subjected to small patient populations, nonrandomization has been adopted in multiple scenarios. Nonrandomized clinical trials that compare against external controls have proven effective in an expanding range of cases, especially in the context of rare diseases. These designs are particularly valuable when randomization is impractical or ethically challenging, or when the available pool of patients with a specific condition is limited [22]. Clinical trial researchers need to understand disease progression and interpret measurements accordingly, but precise methods often don’t translate well to real-world practice. Control groups in these studies are generally being replaced with historical controls based on natural history data. Natural history studies provide a context for a “dry run” of clinical trials, facilitate biological endpoint selection, help in designing an informed clinical trial program with appropriate inclusion/exclusion criteria, help in selecting proper biomarkers for treatment delivery [28], and help to understand long-term trial issues. Errors in this stage are less costly and can inform improvements in the actual trial. Clinical assessments are performed accordingly under small patient population settings providing contextual evidence for regulatory assessment. Incorporating external controls in clinical trials requires meticulous analysis and adept adjustment. Controls are chosen from data obtained from registries, medical records, and scientific literature. Data are also obtained from data from expanded access programs, which provide access to investigational treatments for patients with serious or life-threatening conditions [22]. The process of utilizing external controls entails rigorous statistical methods that help minimize potential biases that can influence results and ensure the validity and reliability of the trial findings.

#### Protocol flexibility for conducting rare disease clinical trials

In many situations, regulators have provided flexibility to sponsors and trial organizers for rare disease clinical trials. Placebo control can be omitted, trial design can be unblinded and nonrandomized, surrogate endpoints can be used for efficacy assessment and trials can be single-arm [3]. Another possible approach is the substitution of clinical endpoints with biomarkers, ideally in the form of a panel of biomarkers representing various aspects of the disease [28] In the case of rare diseases with insufficient biomarkers, the totality of trends in clinical efficacy and safety data can be assessed by utilizing the entire body of available evidence followed by response simulations from clinical trials and pharmacological modeling of data based on reliable biomarkers [25]. These enable adjusting sample sizes, treatment arms, or endpoints, to ensure the most efficient use of resources and maximize the chances of obtaining meaningful results. The preference or requirement for evidence about safety from RCTs must be still present, as these are based on feasibility considerations, such as the practicality of measuring and monitoring safety levels. However, small population trials may not provide sufficient information about safety or efficacy in the long term. To address the unmet medical needs of patients and prioritize public health, it may be possible to consider granting marketing authorizations with less comprehensive data than typically needed [8]. Hence, ongoing monitoring and data collection of a new medicine is being proposed for evaluating efficacy endpoints. It involves monitoring safety, minimizing risks, conducting additional studies for more knowledge, and evaluating risk-minimization strategies. This helps gather valuable insights to enhance understanding and management of the medicine’s benefits and risks [31]. Regulators can provide the flexibility to sponsors to devise a plan for data monitoring and continuous feedback rather than solely relying on trial outcomes. Additionally, protocol flexibility facilitates the inclusion of novel trial methodologies, such as basket or umbrella trials, which can evaluate multiple treatments or subgroups within the rare disease population simultaneously [35]. By embracing protocol flexibility, researchers can address rare disease trial-specific challenges, apply statistical models for design and outcome analysis that are tailored to adaptive trial settings, and improve the chances of successful outcomes and the development of effective therapies for patients with rare diseases. Another aspect regarding adopting flexible protocol is to undertake a multi-faceted approach to train assessors in data evaluation for rare diseases, to enable them to approve applications based on limited data. This includes a deeper understanding of disease heterogeneity, an understanding of the uniqueness of disease manifestation through direct patient interaction, the adoption of machine learning and iterative approaches in data assessment, and ongoing skill enhancement programs. Specific training programs in line with the National Institute of Health (NIH) funded R25 Rare Disease Clinical Research Training Program can impart specialized skills to assessors in rare disease clinical data assessment. To promote innovation in clinical trial design, the Scientific Advice Working Party (SAWP) under the Committee for Medicinal Products for Human Use (CHMP) of the EMA administers a process to evaluate and qualify innovative methodologies for drug development. This involves providing scientific advice and opinions on various methodologies, such as the multiple comparison procedure modeling, which is recognized as an effective statistical approach for model-based design and analysis [8]. 

#### Data extrapolation for efficacy assessment and re-usage of existing treatment

Data extrapolation can be adopted by researchers by leveraging available data from various sources. This can include using historical control data, real-world evidence, registries, electronic health records, clinical literature, and scientific papers or data from similar diseases or patient populations and applying extrapolation techniques to infer the treatment’s effectiveness. By extrapolating data, researchers can make informed judgments about the efficacy of the treatment in the context of the rare disease [36]. This has been utilized to extend existing treatments in adults to the pediatric population based on scientific evidence and assessing their efficacy. This strategy was adopted as part of a need to address the practical and ethical challenges associated with conducting clinical trials in pediatric patient populations and was discussed in an EMA concept paper. If there are scientifically robust data available for an orphan indication, it might be possible to extend these data to offer substantiating evidence for the reasonable certainty of effectiveness, likely advantage, and safety for another orphan indication or subset of a population. The determination of the amount and reliability of data to be utilized for extrapolation, along with the timing of the extrapolation (whether in early or late phase trials), must be carefully assessed on an individual basis for each case [16]. Data can be obtained from clinical trials, real-world evidence, and non-clinical studies. Early identification of relevant data, in collaboration with regulatory authorities, is crucial and can be aligned with a pediatric investigation plan development during initiating studies in adults [23]. As there is limited experience and educational gap in this area thus far, it is important to assess all the nuances and plan the methodologies and validation models by applying rigorous statistical and scientific methods to ensure the correctness and reliability of the extrapolated results. To address knowledge gaps and skills, it is crucial to implement a skill development initiative and continuous assessment for biostatisticians, clinicians, and data analysts.

#### Engagement of patients during study design

It is important to engage the currently known patient population through patient advocacy groups, outreach programs, and advertisements while designing clinical trials. Engaging patients at an early stage and continuous feedback and information sharing can help understand the concerns of the relevant group and can streamline various aspects of trial design, such as safety considerations, benefit-risk assessment, and selection of endpoints [16]. It is recommended to consult with patients who have experience with clinical trials, and early engagement is preferable. Study designs that are based on patient preferences can serve as crucial and valuable reference points in the overall drug development process and regulatory decision-making. Patient motivation also plays a crucial role, as any new treatment that can prove to be effective will have increased interest among target groups and continuous engagement gives a sense of responsibility and belonging to the overall process [5]. Patient participation and retention are very crucial in conducting successful orphan drug trials, especially in countries with limited patient populations. Collaboration across international centers, engagement with patient advocacy groups, involvement of specialist centers, and proper patient education by research staff is essential for successful participation [8]. 

#### Use of Real-World Data in rare disease trial design

Clinical trials face numerous challenges that hinder the timely delivery of promising treatments to patients. However, the use of real-world data (RWD) is emerging as a tool to improve the pace of therapy development. RWD, collected during routine patient care, patient-reported outcomes, handwritten notes, medical records, charts, electronic health records, registry data, and observer reports is gaining support from regulators and can be applied in three key use cases: synthetic control arms (SCAs) to replace or augment standard control arms in trials, precision registries for adaptive trial design, and clinical trial site feasibility to improve patient enrollment. SCAs based on a patient’s standard of care (SOC), in particular, offer an alternative to traditional control arms, which may be unethical in certain cases and help in the identification of endpoints that may vary based on the patient’s disease condition [37]. RWD also enables researchers to optimize study design, assess site feasibility based on patient demographic information of the site, collect more comprehensive and diverse data, and overcome challenges associated with data quality through the application of statistical methods and machine learning algorithms [38]. The integration of RWD in clinical trials holds promise for advancing the development of therapies by quantifying benefits or risks, thereby increasing the success probability and improving health outcomes for patients [23]. The key challenges lie in ensuring data quality, reliability, and interoperability. As many RWDs are extracted through electronic means there are chances of missing data points, and the quality of data may become compromised when merging with multiple other sources for analysis purposes. It is important to classify the data based on type and source and apply appropriate extraction and transformation procedures before they are analyzed for decision-making. To improve interoperability and to ensure data privacy, tokenizing clinical data will be helpful enabling intersystem operability, reliability of information, and availability and accessibility as and when needed [39]. RWD leads to the generation of appropriate RWEs that enable hybrid study methodologies combining the strengths of RCT studies with RWE such as RWE-RE (Real World Evidence-Randomized Enrichment design), mitigating limitations and potentially enhancing or replacing RCTs in many cases, thus providing a patient-centric approach towards statistical data analysis and supporting regulatory decision making [23].

Some steps taken by the International Council on Harmonization (ICH) are to update the E8 and E6 guidelines to include more flexible study designs and diverse data sources. This includes discussions on pragmatic study designs and guidance on using RWD in conjunction with or as a substitute for traditional data collection. The Professional Society for Health Economics and Outcomes Research [40]–International Society for Pharmacoepidemiology [41] (ISPOR-ISPE) Special Task Force has also provided recommendations to enhance clinical study practices and improve the acceptance of RWE by regulatory authorities, emphasizing good practices, transparency, and overall study conduct [42]. As expertise grows, researchers, clinicians, and regulators are likely to adopt and apply these designs on a larger scale [22]. Since the publication of the Operational, Technical, and Methodological (OPTIMAL) framework in 2019, there certain initiatives to incorporate selective usage of RWE for informing regulatory decisions by EMA.

#### Innovative approach to drug development planning

The development planning of rare disease trials necessitates a unique approach. Most of the companies still adhere to traditional clinical pharmacology approaches, which may not be as effective for rare diseases due to their unique characteristics and the small patient populations involved [43, 44]. As a result, many companies are adopting the oncological approach, where the product is tested in Phase I, particularly in patients with high morbidity or mortality. This approach allows the enrolment of a substantial number of patients and enables early assessment of the product’s safety and efficacy. This further helps in the reduction of overall trial costs and helps in resource optimization [45]. The data findings facilitate Model Informed Drug Development (MIDD), to enable addressing gaps in pharmacological studies and provide sufficient clinical data to initiate later phases of trials. This helps in better clinical evaluation and facilitates dose adjustments as well as endpoint modifications across different patient subpopulations. This approach also ensures that high-risk patients are not subjected to exclusion based on randomization and receive the treatment as part of the standard of care procedures [44, 45]. 

Small to medium-sized enterprises (SMEs) are integral to the development and distribution of treatments for rare diseases. These companies play a crucial role in optimizing logistics and leveraging expertise [46]. They often enter into commercial agreements with larger companies, facilitating initial developmental activities of the target therapy, performing an initial assessment of the product’s safety and efficacy, and transferring the same to larger entities for carrying out later activities. Through collaborative efforts, SMEs can access the resources and knowledge of larger companies, potentially accelerating the development and approval of treatments efficiently and effectively [47, 48]. This collaborative model can help to overcome the challenges associated with developing treatments for rare diseases, facilitating reduced timelines, and optimizing the financial and resource burdens, ultimately benefiting patients who may not have access to these life-saving therapies otherwise [47, 48]. 

#### Clinical trial research initiatives

Under the Seventh Framework Program (FP7), the European Union [2] funded three research projects to identify innovative trial designs for rare diseases. The challenges around small patient populations and heterogenous endpoints are recognized by researchers, industry, and regulators and these initiatives aimed to address these challenges and develop effective study methodologies and strategies to address these challenges. The studies aimed to improve the statistical methodology employed in trials involving a small number of patients through enhanced focus on the integration of trial design, conduct methodology, and outcomes and endpoint analysis. This approach ensures a comprehensive perspective and leads to refined methodologies that optimize the overall trial process [8]. The three projects have contributed to the implementation of study models and frameworks that are being increasingly adopted by researchers and sponsors to conduct rare disease clinical trials. Regulators have also shown increasing acceptance of novel methodologies and incorporating the findings in orphan drug approvals. More such initiatives focusing on specific therapeutic areas in coordination with ERNs will help in the development of other design methodologies that may address time criticality, can address endpoint adjustments, and modify the design based on continuous patient feedback. One key initiative that can be taken is a program similar to the Rare Disease Endpoint Advancement Program (RDEA) [49] by the US Food and Drug Administration (FDA). This is a dedicated program to advance the development of endpoints for clinical trials in rare diseases. This can be an EC-funded program under the supervision of EMA in collaboration with researchers, sponsors, and other regulatory authorities focusing on identifying and creating innovative, meaningful, and reliable endpoints to effectively assess rare disease treatment effectiveness. The EC-funded projects are listed below in Table 2:

**Table 2:** FP7 Projects for Improving Small Population Clinical Trials

Project Name	Description
IDEAL: Integrated Design and Analysis of small population group trials [50]	Framework to enable novel, efficient, and effective study design and analysis for clinical trials involving small patient populations. It aims to optimize the development of therapies for rare diseases or specific patient subgroups
InSPiRe: Innovative methodology for small populations research [51]	Create innovative methodologies for designing and analyzing clinical trials in small populations and addressing specific challenges in four areas: • Conducting dose-finding studies in small populations during early phases • Implementing decision-theoretic approaches for clinical trials in small populations • Conducting confirmatory trials in small populations and personalized medicine settings • Using a synthesis of clinical evidence to plan and interpret clinical trials for rare diseases in small patient populations [52].
ASTERIX: Advances in Small Trials Design for Regulatory Innovation and excellence [53]	Offer validated and innovative design methodologies based on statistical models for cost-effective clinical trials conducted with a study having a small number of patients, with a particular focus on rare diseases. This will ensure reliable results and optimize the utilization of resources in these trials.

## Conclusion

The development of effective treatments for rare diseases remains a significant challenge due to the low prevalence and unique characteristics of these conditions. The challenges faced in clinical trials for orphan drugs in the EU are multifaceted and include patient recruitment, disease heterogeneity, limited knowledge of the natural history of rare diseases, lack of existing clinical study data, variability in disease characteristics, ethical considerations, cost challenges, and regulatory hurdles. Despite various financial incentives and grants extended to sponsors and researchers for rare disease research, all these challenges prove to be significant roadblocks in drug development as efficacy assessment becomes difficult due to the lack of test subjects and insufficient data that does not give enough evidence in favor of the medicine. Hence, close attention is required on an ongoing basis to address these issues. Due to the unique nature of rare diseases, there may not be a fit-for-all approach and traditional randomized trial methodologies prove ineffective in different settings. To mitigate these challenges, a few steps have been and can be taken, including the establishment of unified patient databases, PROMs, and RWE generation that has provided and has further potential to provide valuable information for drug development. Innovative approaches to clinical trial design, such as adaptive trial designs, basket and umbrella trials, and the use of real-world evidence, are being explored to address the challenges associated with rare disease trials. Apart from these, by embracing innovative approaches to drug development planning, utilizing external natural history study-based controls, using statistical modeling based on registry, standard of care, and natural history data, ensuring protocol flexibility, engaging patients, and incorporating RWD, researchers can overcome the unique challenges of rare disease trials and improve the development of effective therapies for patients with rare diseases. Continued collaboration between stakeholders, including researchers, clinicians, regulators, patient advocacy groups, and industry is required to foster continuous innovation and the proposed approaches aim to improve the efficiency, effectiveness, and generalizability of clinical research in the context of rare disease patient populations.

### Electronic supplementary material

Below is the link to the electronic supplementary material.

Supplementary Material 1

Supplementary Material 2

## Data Availability

All data used for analyzing the clinical trial landscape were accessed from the International Clinical Trials Registry Platform (ICTRP) of the World Health Organization. The data and related information are publicly available at: https://trialsearch.who.int/. All information generated during this study is presented and documented within the article itself. The data used and analyzed in the study is publicly available in Supplementary File 2. This includes all the necessary information and relevant data points used in the study.

## Abbreviations

CHMP: Committee for Medicinal Products for Human Use
COA: Clinical Outcome Assessment
DCT: Decentralized Clinical Trial
DHT: Digital Health Technology
EC: European Commission
EJP-RD: European Joint Program on Rare Diseases (EJP-RD)
EMA: European Medicines Agency
ERDRI: European Rare Disease Registry Infrastructure
ERICA: European Rare Disease Research Coordination and Support Action Consortium
ERN: European Reference Network
EU: European Union
EUCOPE: European Confederation of Pharmaceutical Entrepreneurs
EURORDIS: European Organization for Rare Diseases
ICH: International Council for Harmonisation of Technical Requirements for Pharmaceuticals for Human Use
IRDiRC: International Rare Diseases Research Consortium
ISPOR-ISPE: Professional Society for Health Economics and Outcomes Research–International Society for Pharmacoepidemiology
NIH: National Institute of Health
OPTIMAL: Operational, Technical, and Methodological Framework
PROM: Patient Reported Outcome Measures
RCT: Randomized Controlled Trial
RDEA: Rare Disease Endpoint Advancement Program
RWD: Real World Data
RWE: Real World Evidence
RWE-RE: Real World Evidence– Randomized Enrichment
SAWP: Scientific Advice Working Party
SCA: Synthetic Control Arm
SOC: Standard of Care
US FDA: US Food and Drug Administration
